# Myelin Organization in the Nodal, Paranodal, and Juxtaparanodal Regions Revealed by Scanning X-Ray Microdiffraction

**DOI:** 10.1371/journal.pone.0100592

**Published:** 2014-07-01

**Authors:** Hideyo Inouye, Jiliang Liu, Lee Makowski, Marilena Palmisano, Manfred Burghammer, Christian Riekel, Daniel A. Kirschner

**Affiliations:** 1 Department of Electrical and Computer Engineering, Northeastern University, Boston, Massachusetts, United States of America; 2 Department of Chemistry and Chemical Biology, Northeastern University, Boston, Massachusetts, United States of America; 3 Division of Cell Biology & Genetics, Università Vita-Salute San Raffaele, Milano, Italy; 4 European Synchrotron Research Facility, Grenoble, France; 5 Biology Department, Boston College, Chestnut Hill, Massachusetts, United States of America; Consejo Superior de Investigaciones Cientificas, Spain

## Abstract

X-ray diffraction has provided extensive information about the arrangement of lipids and proteins in multilamellar myelin. This information has been limited to the abundant inter-nodal regions of the sheath because these regions dominate the scattering when x-ray beams of 100 µm diameter or more are used. Here, we used a 1 µm beam, raster-scanned across a single nerve fiber, to obtain detailed information about the molecular architecture in the nodal, paranodal, and juxtaparanodal regions. Orientation of the lamellar membrane stacks and membrane periodicity varied spatially. In the juxtaparanode-internode, 198–202 Å-period membrane arrays oriented normal to the nerve fiber axis predominated, whereas in the paranode-node, 205–208 Å-period arrays oriented along the fiber direction predominated. In parts of the sheath distal to the node, multiple sets of lamellar reflections were observed at angles to one another, suggesting that the myelin multilayers are deformed at the Schmidt-Lanterman incisures. The calculated electron density of myelin in the different regions exhibited membrane bilayer profiles with varied electron densities at the polar head groups, likely due to different amounts of major myelin proteins (P0 glycoprotein and myelin basic protein). Scattering from the center of the nerve fibers, where the x-rays are incident *en face* (perpendicular) to the membrane planes, provided information about the lateral distribution of protein. By underscoring the heterogeneity of membrane packing, microdiffraction analysis suggests a powerful new strategy for understanding the underlying molecular foundation of a broad spectrum of myelinopathies dependent on local specializations of myelin structure in both the PNS and CNS.

## Introduction

Myelin, which is elaborated by Schwann cells in the peripheral nervous system (PNS) and by oligodendroglial cells in the central nervous system (CNS), constitutes a high resistance, low capacitance, multi-lamellar spiral wrapping of membranes around the axons of nerve cells, and accounts for the substantial increase in nerve impulse conduction velocity compared to that in non-myelinated nerves [1]. X-ray diffraction studies of myelin have provided an abundance of information about the distribution of lipids and proteins perpendicular to the membrane plane [2], [3], [4]. By characterizing the differences between CNS and PNS myelin, differences among species [5], and changes due to neuropathies [6], [7], these studies have also provided detailed structure-function correlates for internodal myelin. This work typically utilized x-ray beams of at least 100–200 µm in diameter resulting in diffraction that represents an average of scattering from all the myelin sheaths within the scattering volume, including numerous axons and their associated nodal, paranodal, and juxtaparanodal specializations [8], [9]. Because most of the volume of myelinated axons consists of internodal (compact) myelin [10], the bulk of structural information produced by x-ray scattering studies to date is predominantly relevant to these multilamellar regions which dominate the scattering.

Internodal myelin at both cytoplasmic and extracellular appositions is stabilized by P0 glycoprotein in PNS myelin and by both proteolipid protein (PLP) and myelin basic protein (MBP) in the CNS [11]. The internode includes interruptions by cytoplasm-containing Schmidt-Lanterman incisures [12] common in PNS, and interlamellar junctional complexes (“radial component”) in the CNS [13]. At the paranode, axo-glial junctions anchor the terminal loops of the ensheathing Schwann or oligodendroglial cell to the underlying axolemma and help to sequester the sodium channels at the node of Ranvier from the potassium channels in the juxtaparanodal region [1]. The axo-glial junctions are morphologically similar to the ladder-like septate junctions [14] and are composed of the axolemma proteins contactin and contactin-associated proteins (Caspr), and glial proteins including neurofascin 155 [15], [16]. Ultrastructural methods reveal that this junction forms a two-dimensional lattice [16]. Dysjunction of the axon-glia adhesion may occur in demyelinating pathologies [17], [18].

In the current study, we explored the structural complexity of myelin within these specialized regions using a 1 µm-diameter x-ray beam. Individual myelinated fibers teased from a mouse sciatic nerve that had been lightly fixed in glutaraldehyde were mapped across the beam with a step size of 1 µm. The diffraction patterns revealed that both paranodal and internodal structural heterogeneities were preserved. In the juxtaparanode-internode region, low-angle reflections from ∼200 Å-period myelin arrays were oriented largely perpendicular to the fiber axis, whereas in the paranode-nodal region ∼210 Å-period myelin arrays were oriented parallel to the fiber axis. Orientation of the lamellar scattering was, therefore, consistent with expectations from electron microscope observations of these differentiated regions of the myelin sheath. In addition, diffuse scattering was observed when the x-ray beam was oriented perpendicular to the membrane planes, e.g., at the center of the nerve fibers, and this was consistent with x-ray scatter arising from the lateral aggregation of P0 molecules. Finally, the electron density profiles of myelin layers in the juxtaparanode-internode showed a variation of electron density at the polar head groups, possibly due to preferential distributions of major myelin proteins in lipid rafts [19].

## Results and Analysis

### Scattering from a pair of overlapping fibers, one including a node of Ranvier

**Figure 1:**
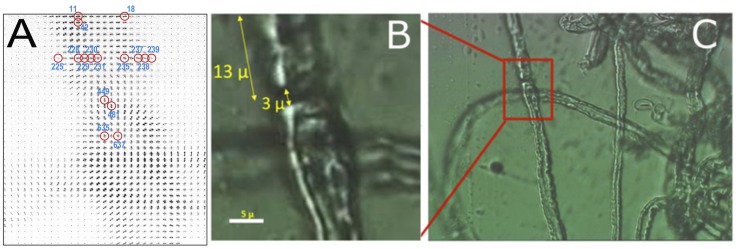
Microdiffraction from single, teased fibers of myelinated axons. (**A**) Montage of the small-angle portions of diffraction patterns from a raster scan of a pair of teased nerve fibers. The vertically-oriented nerve has a node of Ranvier slightly above its crossing with the horizontally nerve. Individual frames in (A), circled and numbered, were chosen for detailed analyses described in this paper. A video of stepping through all of the images in (A), with a white dot in the last frame for each row, is available as Fig. S1. (**B**) Optical micrograph of the same field of view as the montage. (**C**) A larger field of view of the nerve fibers.

**Fig. 1A** is a composite of 1086 diffraction patterns taken from the mesh scan. As can be readily appreciated from the accompanying video (**Fig. S1**), the intensity and orientation of scatter in these patterns varied systematically across the mesh and corresponded to the molecular architecture underlying the features of the sample that were observed in the optical micrograph of the same and larger fields of view (**Fig. 1B, C**). The regions of greatest total intensity correspond to positions where the incident beam impinges the fiber at its periphery, giving rise to strong lamellar scattering due to the high electron density contrast across the width of the membrane. Positions where the periphery of both nerves was irradiated exhibited two principal sets of lamellar reflections nearly perpendicular to each other. The much weaker intensity exhibited from patterns taken near the central region of the nerve fiber indicated that the contrast in electron density in the plane of the membranes is relatively low, and proteins that give rise to most of that contrast are randomly distributed in the membrane plane. Other variations in scattering reflect differences in molecular architecture in the regions adjacent to the node. The large variation in scattering among patterns can be seen in the particular examples detailed below.

To test for possible radiation damage owing to the intense x-ray beam, we recorded a series of 200-msec long diffraction patterns from the same position in the internode. There was no decrease in intensity for at least 2 sec (**Fig. S2**), indicating that there was little or no radiation damage.

Detailed analysis of the individual diffraction patterns constituting the scanning microdiffraction mesh required automation of analytical methods that are conventionally carried out on one diffraction pattern at a time. To provide an overview of the variety of scattering patterns observed and the distribution of their diverse characteristics across the mesh, we developed algorithms to derive a few simple properties for each pattern and mapped these properties onto a corresponding grid. Individual patterns within the grid were selected for detailed analyses.

### Position of the highest intensity in each pattern

**Figure 2:**
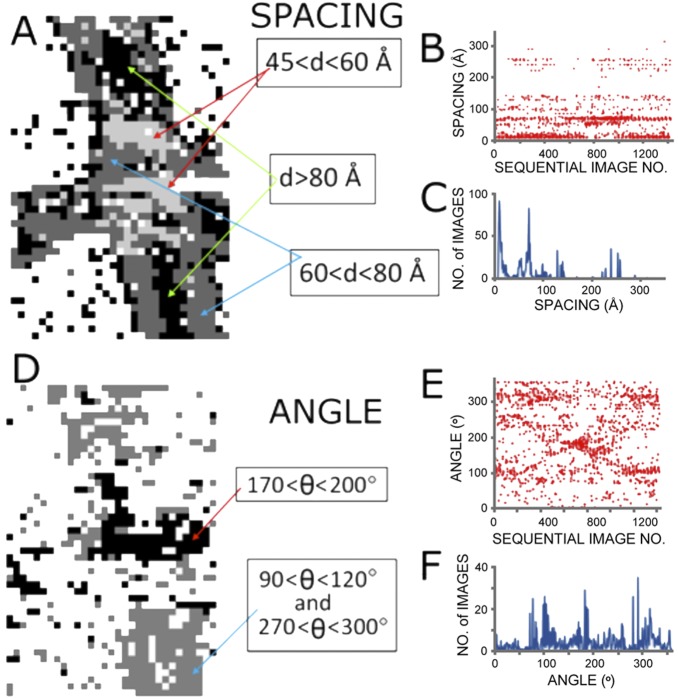
Principal x-ray spacings and their orientations correspond to morphologically distinct regions. (**A–C**) Spacing *d* and (**D–F**) orientation θ for the x-ray reflection having the greatest intensity in each diffraction pattern for the raster-scan of Fig. 1. (**A**) Mapping of position for the different spacings by gray level. (**B**) Scatter plot of the different spacings as a function of sequential image number. (**C**) Histogram of the data shown in (B). (**D**) Mapping of position for the different orientations. Black indicates lamellae with membrane planes approximately horizontal; gray indicates lamellae with membranes approximately vertical on the page. (**E**) Scatter plot of orientation as a function of sequential image number. (**F**) Histogram of the data shown in (E).

The position of the highest intensity of x-ray scattering in each pattern was identified and the corresponding Bragg spacing and angle about the center of the diffraction pattern were determined. Histograms of their values provided guidance for defining clusters of the most common values for these parameters and their distribution across the grid (**Fig. 2**). A scatter plot of the spacing corresponding to the highest intensity exhibited four principal clusters (**Fig. 2B, C**) as represented by different gray levels corresponding to *d*>80 Å (black), 60<*d*<80 Å (dark gray), 45<*d*<60 Å (light gray), and *d*<45 Å (white) that were mapped onto the image grid (**Fig. 2A**). These four clusters corresponded to morphologically distinguishable regions of the fiber. The paranodal-nodal region was dominated by patterns with maxima in the range 45<*d*<60 Å, corresponding to the 4^th^ order of x-ray scatter from the myelin lamellae. Internodal regions were dominated by scattering that had the greatest intensity in the range 60<*d*<80 Å, typically the 3^rd^ order lamellar reflection, which suggests that the molecular organization of the multilamellar myelin in the internodal regions was different from that of the paranodal-nodal region. Finally, regions that had a maximum intensity at *d*>80 Å were most often in the central portion of the fiber (corresponding to face-on scattering, detailed below). This scattering is most likely associated with the lateral organization of proteins in the plane of the membrane, but could also be associated with the 2^nd^-order reflection of lamellar scattering from stacks of myelin membranes.

The angle of maximum intensity was similarly analyzed and mapped (**Fig. 2D–F**). Orientations at two nearly perpendicular angles dominated: one in the range 90°<θ<120° or 270°<θ<300° (**Fig. 2D** **, light gray**) and the other in the range 170°<θ<200° (**Fig. 2D** **, black**). The former feature mapped to membrane lamellae wrapping the vertically-oriented nerve fiber and the latter mapped to the region of overlap with the horizontally-oriented nerve.

**Figure 3:**
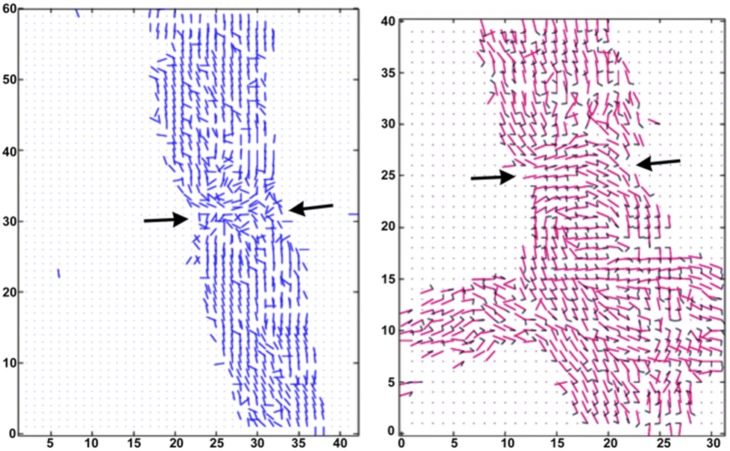
Orientation of patterns reveals changing in wrapping of myelin around axon. Mapping of the principal orientations of lamellar membranes in the region of a node of Ranvier (*arrows*) for a single myelinated nerve (***left***) and a pair overlapping fibers (***right***). To determine the overall orientation of the membrane planes on the nerve fiber, at each position we drew a line parallel to the plane of the membranes and perpendicular to the membrane stacking. The vast majority of reflections in the internode corresponds to membranes oriented parallel to the surface of the fiber. Neighboring the node of Ranvier, however, the lamellar stacks curl around and become more perpendicular to the axis of the nerve fiber (*arrows*). For clarity, orientation of less intense lamellar scattering is not shown for the single fiber (*left panel*). On the right, the orientations of the two most intense sets of lamellar reflections are shown by long red and short blue lines, respectively. Detailed analysis in this paper focuses on the image to the right.

**Fig. 3** shows the distribution of the orientation of the principal lamellar reflections onto the meshes from two different raster scans, one from a single fiber and the other from the overlapping fibers (left and right panels, respectively). The orientation of each diffraction pattern, which is represented by a single line parallel to the membrane planes, shows how the membrane wrapping shifts from being directed around the long axis of the fiber in the internodal region to being perpendicular to the fiber at the node of Ranvier. Moreover, as discussed below, some diffraction patterns exhibited multiple sets of lamellar reflections at different angles about the center of the diffraction patterns, indicating the presence of two (or more) lamellar stacks of membranes rotated relative to one another.

### Scattering from the internodal region

**Figure 4:**
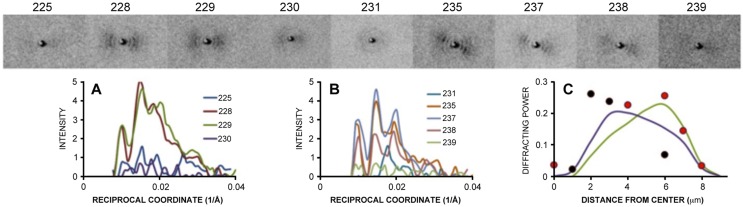
Diffraction patterns across fiber breadth demonstrate lamellar versus in-plane scatter. (***Above***) Diffraction patterns at beam positions #225, 228, 229, 230, 231, 235, 237, 238, and 239. (**A, B**) Observed intensity distributions as a function of reciprocal coordinate (1/Å) for patterns #225, 228, 229, and 230 (**A**), and for #231, 235, 237, 238, and 239 (**B**). (**C**) Diffracting power (defined in **Text S1**, *Diffracting power of myelinated fiber*) as a function of the distance from the center for the observed patterns graphed in panel (**A**) (*black circles*) and in panel (**B**) (*red circles*), and calculated values based on the geometry of the fiber and beam, and where the beam is incident on the fiber (see **Fig. S3**).

Nine diffraction patterns representing positions spanning a cross-section of the vertical nerve fiber in **Fig. 1** (panel A, patterns #225, 228, 229, 230, 231, 235, 237, 238, and 239) were chosen for detailed examination (**Fig. 4**). Lower scattering was observed for the patterns at the left and right edges (#225 and #239) and at the center of the fiber (#230 and #231). Whereas patterns #225 and #239 can be taken to represent the background scatter obtained from this sample, the weak scatter from patterns #230 and #231 likely comes from the distribution of electron density in the plane of the myelin membranes. The stronger intensities observed in the other patterns (#228, 229, 235, 237, 238) originate from lamellar scatter toward the edges of the fiber and are enhanced due to the geometry of the x-rays impinging on the edge rather than on the face of the stacked membranes. Altogether, the distribution of intensity for these nine patterns can be accounted for by a model of the diffracting power expected from a nerve cell with circular cross-section (**Fig. S3**) having an inner radius *r_i_* = 2 µm and outer radius *r_o_* = 8 µm (**Fig. 4C**).

### Myelin membrane structure in the juxtaparanode-internodal region

**Figure 5:**
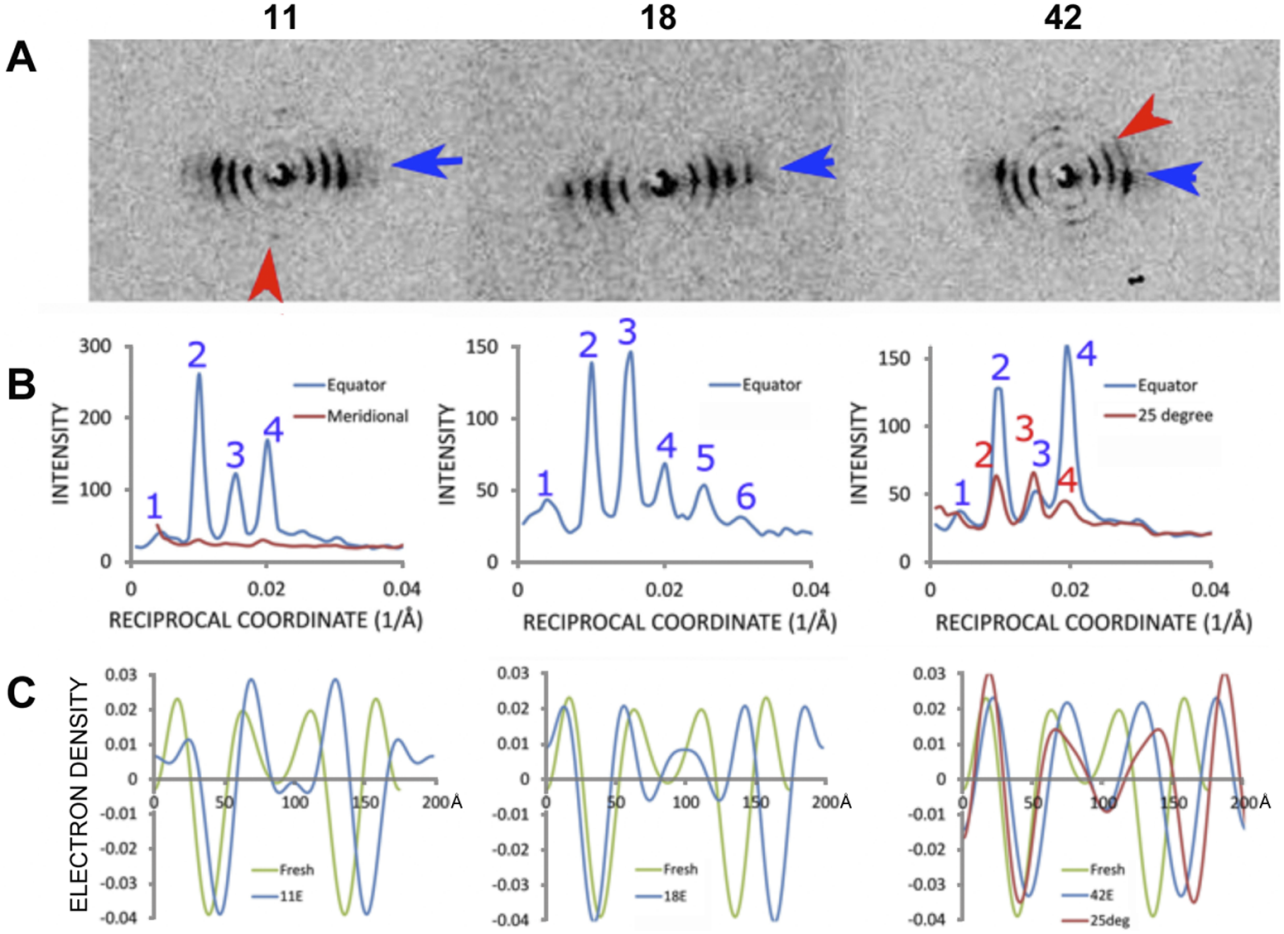
Variation in diffracted intensity demonstrates structural heterogeneity of myelin. (**A**) Diffraction patterns of the internodal regions at positions #11, 18, and 42. Blue and red arrows indicate, respectively, the major and minor reflections. (**B**) Intensity distribution as a function of reciprocal coordinate (1/Å) for the x-ray reflections indicated by the blue and red arrows. Note that in #42, there is also highly-discrete but weak scatter in the vertical direction. (**C**) Electron density distributions (relative scale) as a function of distance (Å) from the center of the cytoplasmic apposition. The myelin periods were 198 Å, 198 Å and 202 Å for patterns #11, 18, and 42, respectively. The data for fresh (unfixed) myelin (green curve), which has a period of 176 Å, was obtained as described in *Materials and Methods (Data Analysis)*.

The juxtaparanode-internodal diffraction patterns exhibited myelin periods in the range of 198–202 Å (**Table 1** and **Fig. 5**), which was about 30 Å larger than typical for fresh nerve tissue. Three patterns in the internodal region were chosen for detailed analysis. Two of these three patterns exhibited a pair of lamellar reflection sets that were orthogonal to one another. The horizontally-oriented reflections (labeled as ‘equator’ in **Fig. 5B**) were uniformly more intense than the vertically-oriented set, indicating that most of the lamellae are wrapped about the nerve cell with the expected geometry. However, an appreciable sub-set of lamellae exist at a significant angle to the primary membrane wrapping (red arrows, #11, #42). In these patterns (#11, 42, and 18), the intensity distributions for the major equatorial reflections (blue arrows) were I(2)>I(4)>I(3)>>I(5)∼I(6) for #11, I(4)>I(2)>I(3) for #42 on the equator (#42E), I(2)∼I(3)>I(4) for #42 of 25 degree tracing (#42 25deg; red arrow), and I(3)>I(2)>I(4)∼I(5)>I(6) for #18. The surprising difference in scattered intensities at neighboring locations emphasizes the structural heterogeneity of the myelin. The coherence length estimated from these patterns corresponded to only about 4–5 unit cells (as calculated from the integral widths of the reflections (**Fig. 5B** and [3]). Assuming that the asymmetric unit of fresh nerve [7] is a close approximation to that of glutaraldehyde-fixed nerve for which the increased periodicity is due to increased membrane separation at the extracellular apposition, the electron density profiles were determined according to our previous analysis of fixed myelin [20].The main differences among the profiles were in the electron density levels of the polar head group layers (**Fig. 5C**). While the electron density exhibited by pattern #42 showed similar electron densities at the polar head group layers to that from fresh nerve, #11 gave much higher density at the extracellular polar head group layer, while #18 gave lower density. Pattern #11 exhibited orthogonal sets of lamellar reflections, indicating the presence of two lamellar stacks of membranes with different orientations; and pattern #42 showed three different orientations of lamellar myelin. By contrast with the predominant set of reflections that was always oriented nearly normal to the fiber axis, the minor set(s) of reflections were along the fiber direction or at an angle to the fiber direction, and were often asymmetric across the center of the diffraction pattern. While the principal myelin scatter in #42 had the intensity distribution I(4)>I(2)>I(3), the minor patterns (oriented at an angle of 25° to 90° from the major lamellar array; red arrow) exhibited a distribution in which I(2)∼I(3)>I(4). The electron density profile calculated from the minor pattern at 25° in pattern #42 (**Fig. 5**) showed a lower electron density at the extracellular polar head group layer and a higher density at the cytoplasmic side compared to the fresh pattern and pattern #42E. These observations suggest that the minor myelin arrays, oriented at an angle to the principal axis of the nerve fiber, have an atypical arrangement of membrane structural components, perhaps related to their proximity to the Schmidt-Lanterman clefts.

**Table 1:** Structural data for characteristic observed diffraction patterns.

	Fresh	11E		18E		42E		42 (25deg)		449M		481M	
**d (h) Å**	174 (5)	198 (6)		198 (6)		202 (5)		205(6)		205 (4)		208 (4)	
**P**	-	54		36		35		26		5		12	
**N**	-	4.4		5.1		4.2		4.1		3.7		4.7	
**Δ**	-	3.3		5.2		5.4		10.1		6.4		9.0	
**u (Å)**	39	47		40		47		43		48		46	
**R-factor**	-	0.28		0.22		0.13		0.25		0.08		0.15	
**Cyt (Å)**	32	48		34		41		37		42		36	
**Lpg (Å)**	47	45		52		54		47		56		59	
**Ext (Å)**	48	60		60		53		74		51		54	
**H**						**F (h)**							
	**Obs**	**obs**	**calc**	**obs**	**calc**	**obs**	**calc**	**obs**	**calc**	**obs**	**calc**	**obs**	**calc**
**1**	−0.148	−0.187	−0.098	−0.230	−0.172	−0.350	−0.111	0	−0.151	0	−0.125	0	−0.166
**2**	1.323	1.031	1.159	0.843	0.947	1.236	1.152	0.673	0.961	1.112	1.199	0.941	1.203
**3**	0.809	1.021	0.352	1.435	1.410	0.565	0.532	1.119	1.200	0.801	0.696	1.412	1.238
**4**	−1.496	−1.656	−1.948	−0.938	−0.862	−1.954	−1.966	−1.258	−1.212	−2.067	−2.052	−1.833	−1.797
**5**	−0.744	−0.464	−0.502	−1.176	−1.488	−0.518	−0.756	−1.514	−1.512				
**6**		0.912	0.644	−0.923	−0.263			−0.751	−0.001				

The structure factors for the intensities measured by the microfocus beam are given by 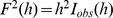, and their scaling was determined by 
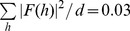
, where *d* is the lamellar period. The diffracting power *P* was measured according to the equation described in (**Text S1**). The intensity data for fresh, mouse sciatic nerve myelin were obtained from Figure 5 in Ref. (7). In the Table, the sample is indicated by the image number, and the scanning direction is indicated by *E*, for along the equator (lamellar stacks perpendicular to the vertical oriented nerve fiber; *M*, for along the meridian (lamellar stacks parallel to the vertically oriented nerve fiber), and *25deg*, for 25° from the horizontal. The distance between the centers of membrane bilayers across the cytoplasmic apposition is given by *2u*. *Cyt*, the width of the cytoplasmic apposition; *Lpg*, the distance between the lipid polar head group layers; *Ext*, the width of the extracellular apposition. The number of unit cells *N*, or coherent length, and lattice disorder Δ were calculated from 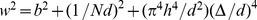, where *w* is the observed integral width for the *h^th^* reflection, and *b* is the integral width of the direct beam [54].

### Paranode-nodal x-ray diffraction

**Figure 6:**
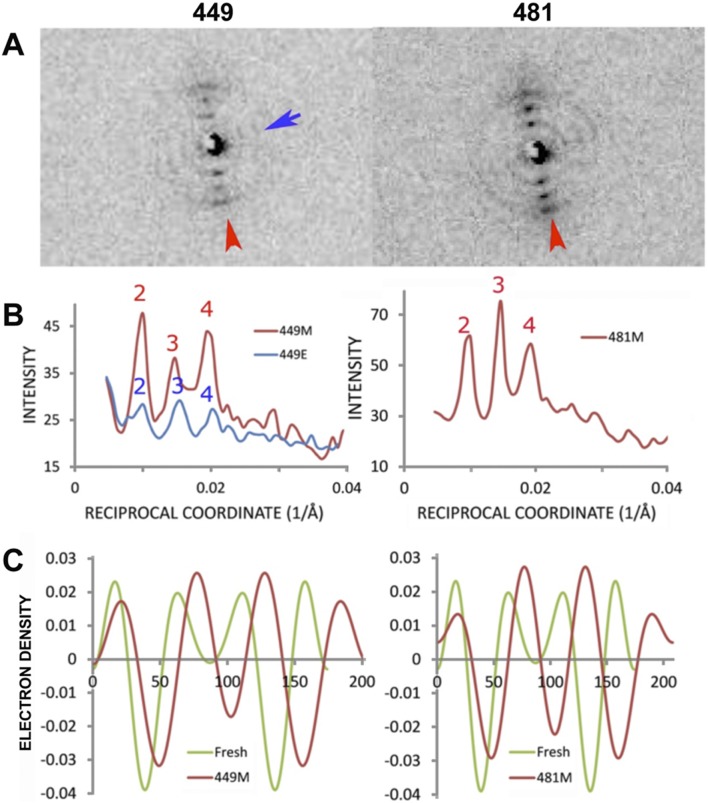
Paranodal-nodal diffraction differs from juxtaparanodal-internodal diffraction. (**A**) X-ray diffraction patterns #449 and #481 from the nodal/paranodal region. (**B**) Intensity distribution (relative scale) as a function of reciprocal coordinate (1/Å), with Bragg orders 2–4 indicated. *M* and *E* refer to meridional (red arrow) and equatorial (blue arrow) scatter, respectively. (**C**) Electron density distribution as a function of distance (Å) from the center of the cytoplasmic apposition. The data for fresh (unfixed) myelin (green curve), which has a period of 176 Å, was obtained as described in *Materials and Methods (Data Analysis)*.

Myelin-like scattering in the nodal regions, exemplified by patterns #449 and #481 (**Fig. 6**), exhibited a repeat period of 205–208 Å. The intensity distributions were I(2)>I(4)>I(3) for #449 and I(3)>I(2)>I(4) for #481 (**Table 1**). The major reflections were oriented parallel to the nerve fiber, indicating that the membrane stacking direction was oriented roughly perpendicular to the fiber axis; and the minor reflections were oriented parallel to the axis. The number of unit cells in the coherent domain was 4–5 which was similar to the estimate for myelin in the juxtaparanode-internode region (**Table 1**). However, the lattice disorder of 6–9 Å was larger than that at the juxtaparanode-internode (3–5 Å). The diffracting power (P in **Table 1**) was smaller than the internodal estimate, indicating that there were fewer membrane arrays. The electron density profiles for the myelin in this region (**Fig. 6C**), calculated as indicated above for fixed myelin, showed that the widths of the cytoplasmic and extracellular appositions were both larger than those for fresh (unfixed) control nerve (**Table I**).

### Scattering from proteins in the plane of the membrane

**Figure 7:**
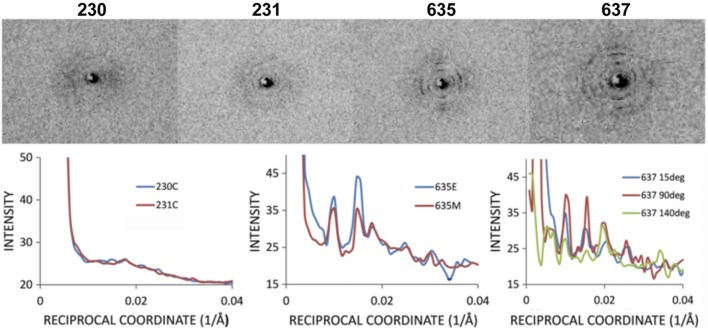
In-plane diffraction attributed to arrangement of protein in membrane surface. (***Upper***) Diffraction patterns resulting from incident x-ray beam *en face* to the myelinated fiber at positions of #230, 231, 635, and 637. (***Lower***) Intensity distribution as a function of reciprocal coordinate (1/Å). *C*, *M*, and *E* refer to circular averaging, and to meridional and equatorial scatter, respectively.

The microdiffraction raster-scan also included patterns that resulted from the incident x-ray beam face-on at the center of the nerve fiber (**Fig. 1A**). For example, patterns #230 and #231 exhibited a broad intensity maximum at ∼57 Å Bragg spacing, and #635 and #637 showed a similar diffuse band and also orthogonal sets of lamellar reflections (**Fig. 7A**). The 57 Å-spacing intensity maximum most likely arises from electron density contrast occurring in the plane of the membranes and may be indicative of the nearest neighbor distance between protein particles in a two dimensional liquid-like arrangement. This intensity was modeled using 2, 3, or 4 solid cylinders with radii of 16 Å to represent the extracellular domain of P0 [21]. When placed on a circle of radius 28 Å to model P0-P0 homophilic interactions [22] these cylinders generated an intensity distribution similar to that observed (**Fig. S4**). The intensity distribution calculated for 2, 3, and 4 molecules of P0 using the atomic coordinates determined by protein crystallography [22] exhibited diffuse scattering in the range of 0.015–0.041/Å (**Fig. S4**) with an intensity maximum at a spacing of ∼40 Å. This is somewhat smaller than observed here and suggests that the separation between the glycosylated full length P0-molecules in myelin of whole nerve is about 10 Å larger than the crystallized non-glycosylated extracellular domain of P0 [22].

## Discussion

### Comparison with previous x-ray diffraction studies of nerve myelin

The nerve myelin sheath has been studied since 1930s by polarized light microscopy, x-ray diffraction [23], [24], [25], and electron microscopy [26]. Extensive x-ray scattering studies of its molecular architecture have led to an understanding of the distribution of protein and lipid in the membranes and the role of those components in myelin assembly, stability, and function [3], [27], [28]. Until recently, the beam sizes used for the x-ray diffraction studies were 100 µm or larger. Because the size of a single nerve fiber is typically 10–30 µm in the PNS, and the axial lengths of the node, paranode, and juxtaparanode regions are 1 µm, 5 µm, and 10 µm, respectively [15], then the 100 µm beam subtends a region that is structurally heterogeneous. In earlier studies, therefore, the observed diffraction is a mixture of scattering from different compartments but dominated by the abundant, compact myelin arrays in the internodal segments [29], [30]. The 1 µm beam size [31] used in the current study was sufficiently small to allow measurement of the scattering from the structurally differentiated regions of the myelin sheath in and bordering the internode. No appreciable radiation damage occurred with this beam, as evidenced by the absence of degradation in Bragg scattering with time (Fig. S2).

Recent synchrotron diffraction studies of myelin (in sciatic nerve and brain) used samples that were either freeze-dried [32] or fixed in formalin [33], [34]. Because dehydration induces a phase separation of myelin into lipid-rich and protein-rich domains having different periodicities [35], freeze-drying is not suitable for preparing the samples for study of structural heterogeneities. By contrast, because it cross-links molecules that are in proximity to one another, glutaraldehyde fixation is likely to preserve the regional diversity of molecular architecture within the myelinated nerves, including the axo-glial junctions [16].

Previous analysis of glutaraldehyde-fixed, mouse sciatic nerves using a 100 µm beam shows a slightly larger period of 178 Å compared to the native 176 Å. The electron density distribution shows that the cytoplasmic separation for the glutaraldehyde treated myelin is smaller than that for the control, while the extracellular separation is larger. Further, the electron density level at the cytoplasmic space is higher than at the extracellular surface, suggesting that the glutaraldehyde crosslinks the abundant lysine and arginine residues [36] in MBP. In the current study, we observed that the myelin periods were 198 Å (patterns #11E and #18E) and 202 Å (#42E) which were much larger, by 22 Å and 26 Å, than that of fresh myelin. The electron density profile of #18 had a cytoplasmic separation (34 Å) that was similar to that of fresh myelin (32 Å), while this apposition measured for patterns #11 and #42 was significantly larger (48 Å and 41 Å). Since these diffraction patterns are all from the internodal region, the differences in the widths of the cytoplasmic appositions apparently reflect local variations in the packing of membranes. We also observed that the electron density level at the cytoplasmic polar head layer was higher than that at the extracellular one for pattern #18 as reported previously [20], while for pattern #11 it was lower; and for #42 it was similar. The apparent correlation between a wider cytoplasmic separation and lower electron density at the cytoplasmic surfaces suggests that the amount of cross-linked MBP may vary spatially in the internode (e.g., lower in the regions generating patterns #11 and #42 compared to the region generating pattern #18).

In the current study, the width of the extracellular separation was larger than that for fresh myelin (i.e., 60 Å for #11 and #18, and 53 Å for #42 compared to 48 Å for fresh myelin). Previous study of glutaraldehyde-fixed myelin [20] also showed swelling at the extracellular apposition, but not to the extent observed here. The absence in the teased single fibers of the mechanical constraint provided by collagen [37] may account for the greater extracellular swellings observed here.

### Deformation of myelin lamellar structure

A surprising finding was the occurrence, in many patterns, of multiple sets of lamellar reflections oriented at different angles to one another. In the internodal region we commonly observed two or more distinct lamellar domains with different orientations. In most cases, the major set of lamellar reflections was oriented as expected, consistent with the wrapping of membrane stacks around the axon. The weaker sets of lamellar reflections correspond to a smaller population of membranes stacked at an angle–usually close to 90°–to the dominant membrane packing direction. These weaker reflections, which were observed in many of the *en face* diffraction patterns, may come from lamellar layers deformed at Schmidt-Lanterman incisures [38], [39]. Interestingly, the intensity distribution in these two sets of lamellar reflections were usually different, indicating that the molecular structure of the principal membrane stacks is different from that of the layers at the Schmidt-Lanterman incisures (see Section 5.4). Other evidence for local deformation comes from the unequal intensity of centrosymmetrically-related reflections (Friedel’s pairs), as for example seen in the vertically-oriented patterns #42, 449, 481, 635, and 637; and this effect probably arises from a highly localized tilting of the membrane stacking.

In many of the patterns from the center of the nerve fibers (where the incident beam is normal or *en face* to the membrane planes), two orthogonal sets of reflections were usually observed. The dominant scattering in these patterns can be accounted for by the arrangement of proteins in the plane of the membrane (see Section 5.3), whereas the weaker sets of reflections may come from lamellar stacks of membranes at unexpected angles as described above. Electron microscopy reveals that the continuous, small pocket of cytoplasm that defines the spiraling Schmidt-Lanterman incisures results in a swelling deformation of the neighboring cytoplasmic space (see review [9]); however, the current x-ray observations suggests a more complex geometry of the deformed myelin layers. Analyzing the orientation and positioning of the reflections recorded in the current study using an even smaller x-ray beam–e.g., 0.5 µm–may enable a more accurate three-dimensional reconstruction of incisures.

### Arrangement of P0 in the myelin membrane plane

Membrane-membrane adhesion at the extracellular apposition in PNS myelin has been studied in order to account for the lamellar stacking defect in dysmyelinated nerves [6], [7], [40]. Membrane separation in myelin appears to be due to long-range electrostatic repulsion and van-der-Waals attractive forces, and also to the short-range head-to-head P0-P0 interactions involving electrostatic interaction between His52 and Arg45 [6]. Membrane-membrane separations at the extracellular and cytoplasmic appositions are also likely to be modulated by laterally-aggregated P0 proteins. While x-ray solution scattering [21], SDS electrophoresis [41], and mass spectrometry [42] indicate that P0 forms dimeric and tetrameric aggregates *in vitro*, aggregation of P0 in intact myelin of whole nerve has not previously been demonstrated. By using an incident microbeam normal to the membrane surface, we observed diffuse scattering with an intensity maximum at 57 Å, which is consistent with that expected from lateral aggregates of P0. We confirmed this by calculating intensity distributions of 2–4 mers of the extracellular domain of P0. The glycosylation of P0 at Asn93, located near the membrane surface [22], may influence P0-P0 lateral interaction, because the observed separation of 57 Å is larger than the 40 Å separation calculated for non-glycosylated P0 molecules.

A broad intensity maximum previously observed at a spacing of ∼50 Å underneath the discrete Bragg orders from multilamellar myelin was interpreted as due to substitution or packing disorder of myelin membranes across the cytoplasmic apposition [43], [44]. Given the similarity of that scatter to the observed diffuse scattering maximum of the *en face,* or in-plane structure reported here, it may be that the previous analyses overestimated the substitution disorder by not considering any contribution from the in-plane organization of protein.

### Molecular interpretation of electron density profile

As indicted above, the intensity distributions from a single diffraction frame from a 1 µm x-ray beam may contain multiple sets of myelin reflections oriented at an angle to one another and having different intensity distributions (see for example, analysis of pattern #42 above). The intensity variation may be accounted for by a difference in protein distribution within the primary multilamellar myelin wrapped around the axons and those presumably distorted at the Schmidt-Lanterman incisures. The segregation of proteins and lipids to these specialized regions may be due to lipid rafts [45] or formation of protein rafts [46]. For example, it has been reported that P0 molecules are associated with glycosphingolipid/cholesterol-enriched membranes [47], and MBP proteins with detergent-resistant membranes (DRMs) [48]. The results reported here showing structural microheterogeneity are consistent with the notion that the major myelin proteins in PNS are not randomly distributed by diffusion in the membrane plane, but may be localized within specific regions.

**Figure 8:**
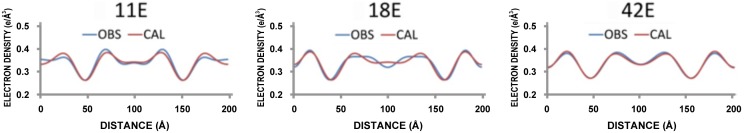
Position-dependent profiles for lamellar myelin consistent with differing ratios of myelin proteins. Electron density distribution on an absolute scale for data from diffraction patterns #11E, 18E, and 42E, where *E* refers to scattering along the equator. The observed and calculated structure factors on a relative scale are indicated in Table 1. (The absolute scaling method is described in *S.I., Section 1.*) The differences in the levels of electron density in the lipid polar head group layers at the cytoplasmic and extracellular sides of the membrane bilayer are interpreted in terms of a heterogeneous distribution of MBP and P0 glycoprotein in different regions of the myelin sheath (see Text for details).

To compare further the scattering from glutaraldehyde-fixed nerve reported here with scattering from fresh nerve (control), we constructed chemical models for myelin and refined the parameters of the models to best fit the observed structure amplitudes on an absolute scale (see calculated structure factors in Table 1). The scale was derived by using the exclusion length of 136 Å, the average membrane electron density within the exclusion length of 0.343 e/Å^3^ and a buffer electron density of 0.335 e/Å^3^ [3], [21]. Comparison of the observed and calculated electron densities (**Fig. 8**) for pattern #11 showed a decrease of 0.015 e/A^3^in the electron density at the cytoplasmic polar head group layer, and corresponding increases in the cytoplasmic region and at the extracellular side. This suggests that the concentration of MBP proteins is lower than in the control, and that the concentration of the transmembrane P0 protein is larger. Since the electron density level in the control myelin of MBPs is 0.04 e/A^3^ and that of P0 is 0.085 at the extracellular domain in the average structure of nerve myelin [3], we conclude that almost half of the MBPs are not localized in this region, at the same time that the P0 content is larger by a quarter relative to that of control. Pattern #18 showed at the cytoplasmic side a similar electron density profile to that of the control, indicating that the contents of MBP and P0 are similar to those of the control. The different electron density level at the extracellular space indicates that the extracellular sides of the P0 molecules are not fully staggered as in the single crystal [22], but slide past one another as in Ser63Cys mutant mouse myelin [6]. Pattern #42 shows an electron density profile similar to control, indicating that glutaraldehyde fixation of the myelin membranes has not dramatically altered the myelin structure.

### Conclusions

We have demonstrated that using a 1 µm x-ray beam to raster-scan an individual, teased nerve fiber can provide unprecedented levels of detail about the specialized molecular architectures of distinct regions of the myelin sheath, as revealed by the spatially-dependent variation in the diffraction patterns. Differences in the membrane separations, distribution of proteins, and the orientation of membrane lamellae can be readily extracted from the data. The richness of information provided by scanning x-ray microdiffraction and the details of molecular organization that this information illuminates suggests that similar approaches using fresh nerve tissue will provide powerful new strategies for understanding the underlying molecular foundation of a broad spectrum of myelinopathies. In particular, progress on instrumentation for the beamline used in our experiments (ID13) can now provide <200 nm beams. The significant increase in lateral resolution in mesh scans will allow a more precise differentiation and identification of neighboring domain structures, not only in myelinated fibers but also in other biological tissues and non-biological materials [49].

## Materials and Methods

The Boston College (BC) Animal Care Program is guided by the ethical principles of research set forth in the *Animal Welfare Act* (7 U.S.C. 2131 et. seq.), *Public Health Service Policy on the Human Care and Use of Laboratory Animals*, The Guide for the Care and Use of Animals, and *National Aeronautics and Space Administration Principles for the Ethical Care and Use of Animals* (1979). BC’s polices and procedures involving animal care and use have been developed to comply with the federal, state and local laws and regulations relating to animals. The research reported here was approved by Boston College IACUC, #2007-002-03; #2011-015.

### Sample preparation

Sciatic nerves were dissected from mice that had been euthanized using CO_2_ inhalation followed by cervical dislocation. Under a dissecting microscope, single fibers from mouse sciatic nerves that had been fixed for 10–30 min in 2% paraformaladehyde-2.5% glutaraldehyde (in 0.12 M phosphate-buffered saline, at pH 7.4) were teased apart with very fine forceps after removal of the perineurium using a 26-gauge, stainless-steel needle. The fibers with a small volume of adhering solution were then aspirated into 0.7 mm-diameter x-ray capillaries, and sealed with wax and fingernail polish enamel.

### X-ray diffraction

X-ray microdiffraction was performed at the ESRF-ID13 beam line. A monochromatic beam of wavelength 1.0 Å was focused to a spot that was 1 µm full-width at half-maximum [50]. The diffraction patterns were recorded using an 16 bit x-ray sensitive MarCCD detector which was 2×2 binned. A single pattern contained 1024×1024 pixels with a 158 µm pixel size. The specimen-to-detector distance was 203.9 mm which was calibrated by the Bragg reflections of silver behenate powder indexed by the fundamental period of 58.38 Å [51]. The exposure time at each position was sufficiently small (200–500 msec) to preclude detectable radiation damage (**Fig. S2**). Mesh scans containing several thousand individual diffraction patterns were recorded for fields that included nodes of Ranvier visualized using a video microscope (see **Fig. 1**).

### Data analysis

Radial intensity distributions were derived by angular averaging the patterns using FIT2D [52], or by straight line tracing across the pattern using ImageJ [53]. For mapping detailed features of the small angle region of the patterns, we cropped 8-bit, 200×200 pixel frames from the original 16 bit, 1024×1024 pixel image using the MATLAB image toolkit (The MathWorks, Natick, MA). The extracted features included the total integrated intensity and Bragg spacings and angles of the pixel position giving the highest intensity within each diffraction frame; and, for selected diffraction patterns, the electron density distribution calculated perpendicular to the membrane plane. For comparison with these electron density profiles, intensity data from fresh (unfixed) sciatic nerve myelin were taken from reference [7]. Details of our analysis of the diffraction patterns are included as **Text S1**.

### Microbeam Scattering from a Myelinated Axon

**Fig. S3** diagrams the geometry of microbeam scattering expected from a myelinated axon calculated for a circular cross-section, assuming concentric wrapping of the myelin membranes about the axon. A microbeam with diameter substantially less than that of the fiber will sample a subset of all the orientations of the membranes. If incident at the center of the fiber, it will generate scattering informative of the electron density contrast in the plane of the membranes. If incident to the periphery of the fiber, it will generate scattering informative about the distribution of protein and lipid across the thickness of the myelin membranes (the classical lamellar scattering from myelinated nerves). Depending on the amount of disorientation within the scattering volume (φ), the intensity of the lamellar scattering will vary substantially as a function of distance from the center of fiber.

## Supporting Information

Figure S1 **Video sequence of stepping through all of the images collected in a single mesh-scan.** An animated succession of the individual diffraction patterns (small-angle region only) for the montage shown in Figure 1(A) clearly shows how the intensity and orientation of scatter varies systematically across the mesh and, moreover, corresponds to the molecular architecture of the myelinated fiber observed in the accompanying optical micrograph (Figure 1B, C). The last frame in each row is indicated by a white dot in the upper right corner.
(AVI)

Figure S2 **Test for radiation damage.** **(A)** The small-angle region recorded for 200 msec per pattern at a single position along the nerve. **(B)** Radial-averaged intensity for the sequentially-recorded patterns. The total time for the 10 patterns was 2.0 sec. The spectra show small variations of intensity, but no overall decrease in intensity, which indicates little or no structure degradation due to radiation damage.
(DOCX)

Figure S3 **Interpretation of scattering from a myelinated nerve.** **(A)** The scattering expected from myelin will be very different if the microbeam hits the fiber in the center (a) versus the periphery (b). The beam hitting the center (a) will be incident *en face* to the membrane faces and small-angle scattering will be informative about the contrast in electron density in the plane of the membranes. When the beam hits the periphery (b), it is incident parallel to the surfaces of the myelin membranes, in which case the scattering is informative about the variation of electron density in the direction perpendicular to the membrane plane, i.e., in the stacking direction. When the incident beam hits the fiber between the center (face-on) and periphery (edge-on) the resulting scatter will be a mixture of the two extreme cases diagrammed in (A). The geometry for a nerve fiber with a circular cross section **(B)** can be used to derive the variation in intensity of lamellar scattering as a function of the distance from the center of the fiber **(C)**. Assuming for the myelin inner and outer radii of 5 µm and 20 µm, respectively, a beam size of 1 µm and different amounts of disorientation (φ) of the membranes in the sample, the predicted intensity distributions are diagrammed in (C).
(DOCX)

Figure S4 **Modeling the in-plane diffraction.** (A) Intensity distribution as a function of radial component of the cylindrical reciprocal coordinate (1/Å) for a dimer (N = 2), trimer (N = 3), and tetramer (N = 4) of solid cylinders with a radius of 16 Å on a circle with a radius of 28 Å [4]. The intensity was normalized so that the area under the curve was one. (B) Cylindrically-averaged intensity distribution as a function of radial component of cylindrical coordinates for the atomic coordinates of the non-glycosylated P0 extracellular domain [9]. Pymol [10] representations of monomer, dimer, trimer, and tetramer of the P0-extracellular domain viewed normal to the membrane surface.
(DOCX)

Text S1 **Myelin Diffraction Analysis.** Details of our analysis of the diffraction patterns are included in the sections titled: *Observed and calculated intensities*; *Phase determination by replacement method*; *Diffracting power of myelinated fiber*; *Concentric multilamellar cylinders*; and *Surface structure and cylindrical averaged intensity calculation*.
(DOCX)
