# Supplementary figures and images for: Myelin Organization in the Nodal, Paranodal, and Juxtaparanodal Regions Revealed by Scanning X-Ray Microdiffraction
Source: PLoS One. 2014 Jul 1;9(7):e100592. doi: 10.1371/journal.pone.0100592 (PMC4077703; doi:10.1371/journal.pone.0100592)

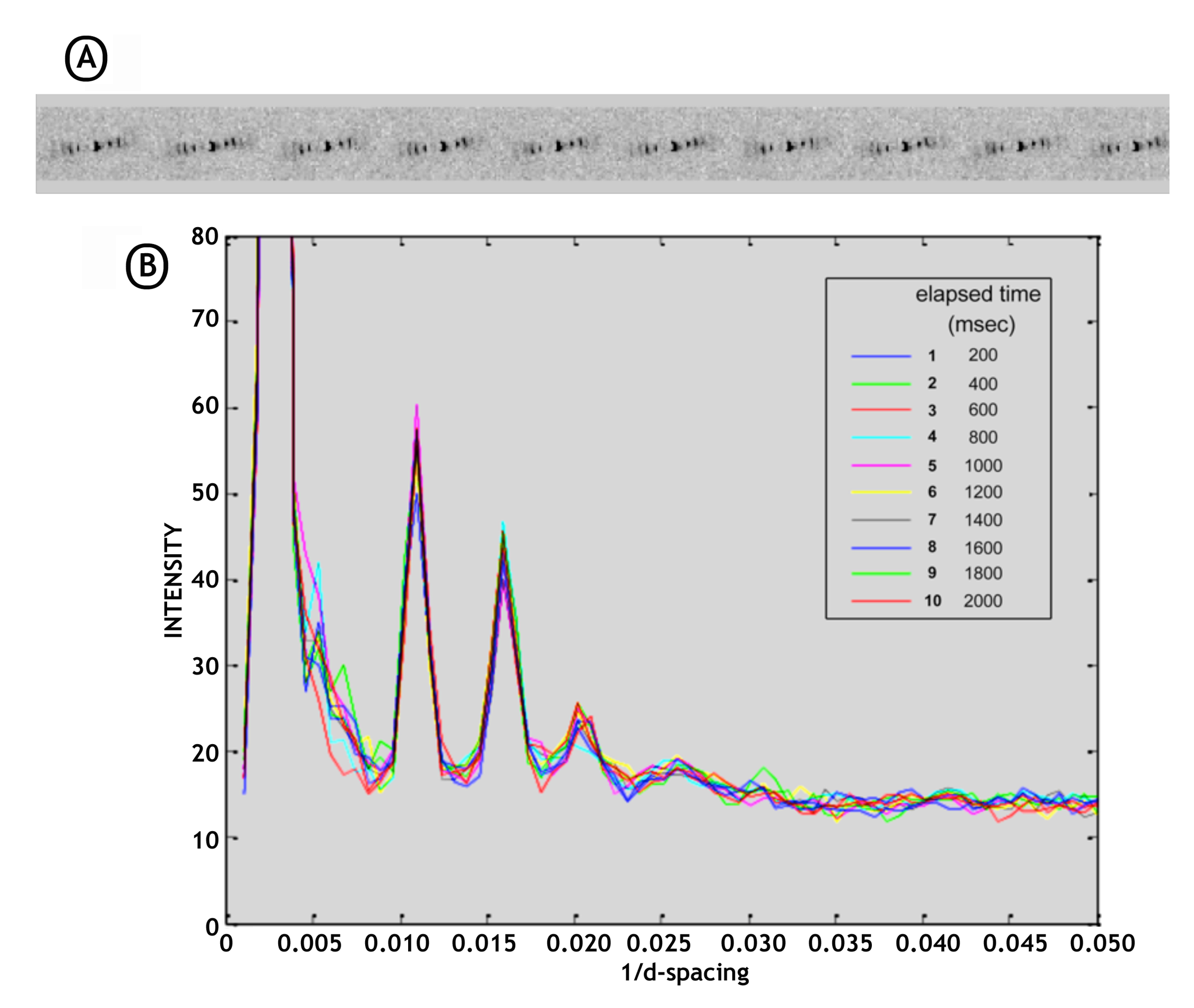

Supplement: Figure S2 — Test for radiation damage. (A) The small-angle region recorded for 200 msec per pattern at a single position along the nerve. (B) Radial-averaged intensity for the sequentially-recorded patterns. The total time for the 10 patterns was 2.0 sec. The spectra show small variations of intensity, but no overall decrease in intensity, which indicates little or no structure degradation due to radiation damage. (DOCX) [file pone.0100592.s002.docx]

**
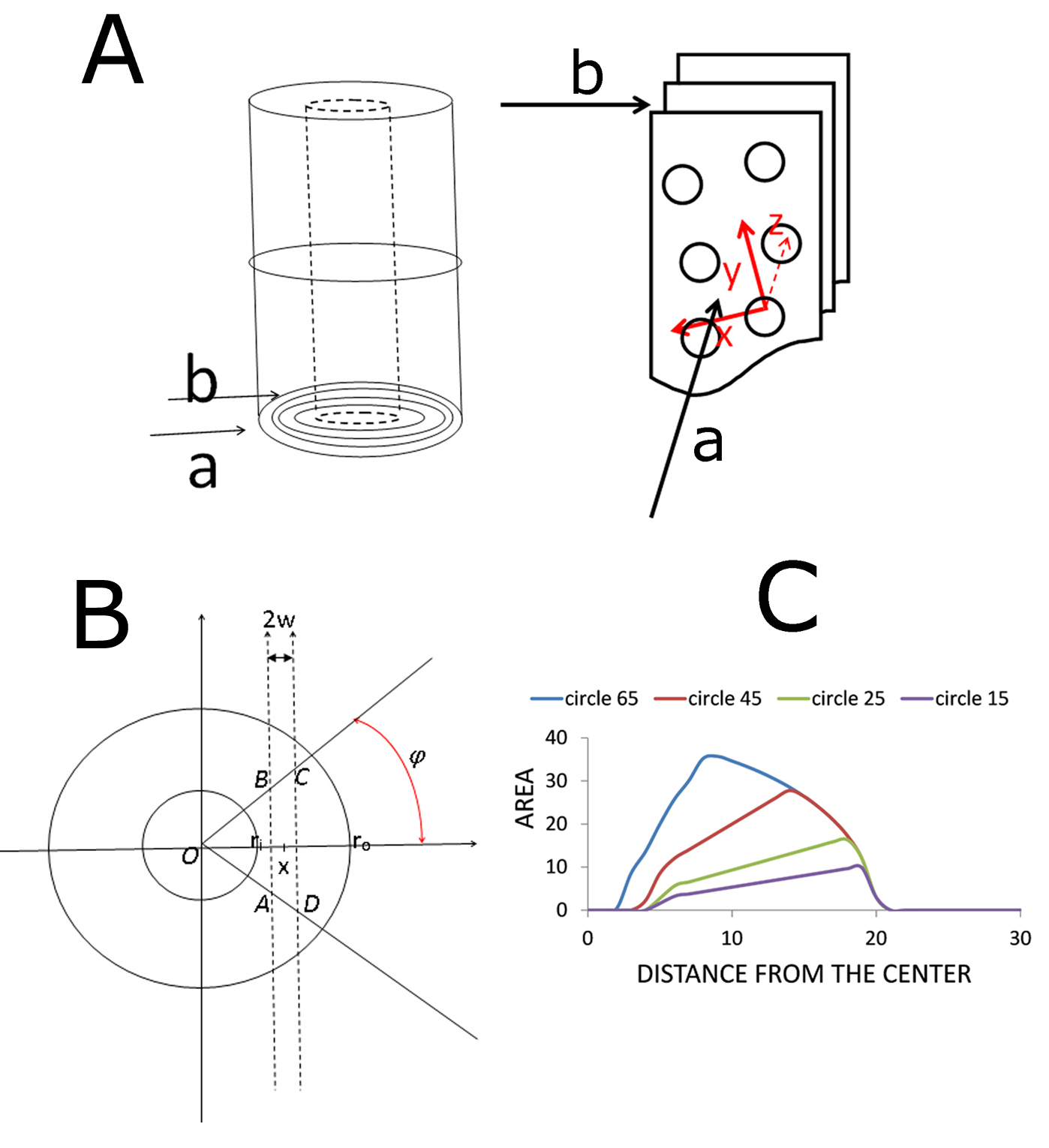
**

Supplement: Figure S3 — Interpretation of scattering from a myelinated nerve. (A) The scattering expected from myelin will be very different if the microbeam hits the fiber in the center (a) versus the periphery (b). The beam hitting the center (a) will be incident en face to the membrane faces and small-angle scattering will be informative about the contrast in electron density in the plane of the membranes. When the beam hits the periphery (b), it is incident parallel to the surfaces of the myelin membranes, in which case the scattering is informative about the variation of electron density in the direction perpendicular to the membrane plane, i.e., in the stacking direction. When the incident beam hits the fiber between the center (face-on) and periphery (edge-on) the resulting scatter will be a mixture of the two extreme cases diagrammed in (A). The geometry for a nerve fiber with a circular cross section (B) can be used to derive the variation in intensity of lamellar scattering as a function of the distance from the center of the fiber (C). Assuming for the myelin inner and outer radii of 5 µm and 20 µm, respectively, a beam size of 1 µm and different amounts of disorientation (φ) of the membranes in the sample, the predicted intensity distributions are diagrammed in (C). (DOCX) [file pone.0100592.s003.docx]

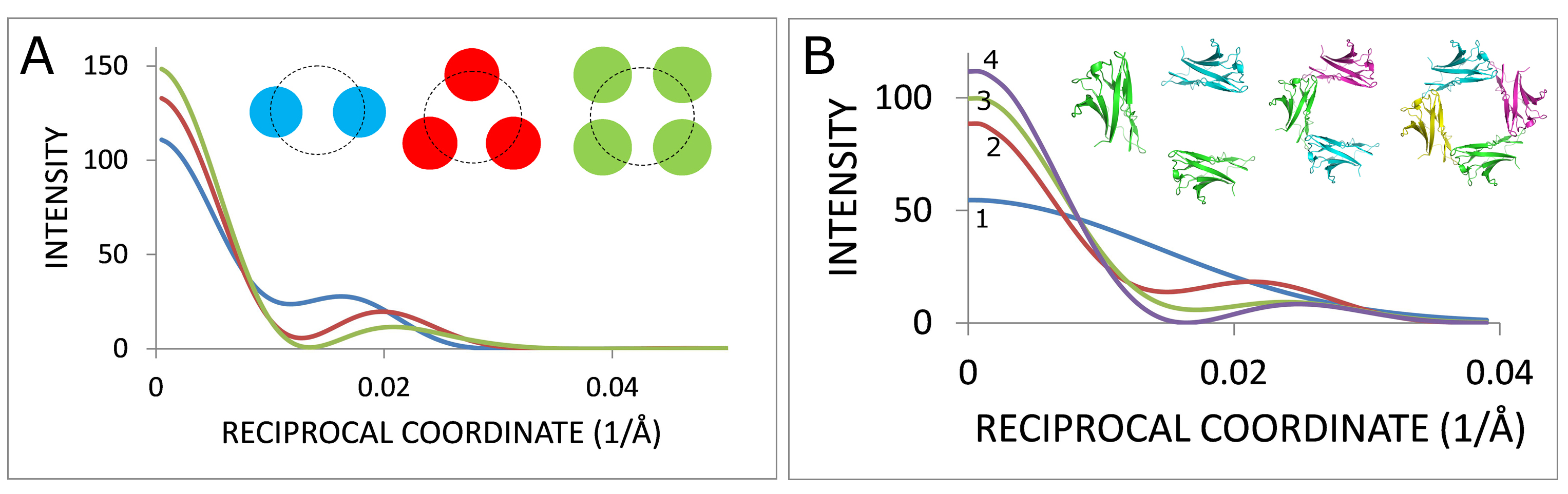

Supplement: Figure S4 — Modeling the in-plane diffraction. (A) Intensity distribution as a function of radial component of the cylindrical reciprocal coordinate (1/Å) for a dimer (N = 2), trimer (N = 3), and tetramer (N = 4) of solid cylinders with a radius of 16 Å on a circle with a radius of 28 Å [4]. The intensity was normalized so that the area under the curve was one. (B) Cylindrically-averaged intensity distribution as a function of radial component of cylindrical coordinates for the atomic coordinates of the non-glycosylated P0 extracellular domain [9]. Pymol [10] representations of monomer, dimer, trimer, and tetramer of the P0-extracellular domain viewed normal to the membrane surface. (DOCX) [file pone.0100592.s004.docx]
