# Supplementary material for: Myelin Organization in the Nodal, Paranodal, and Juxtaparanodal Regions Revealed by Scanning X-Ray Microdiffraction
Source: PLoS One. 2014 Jul 1;9(7):e100592. doi: 10.1371/journal.pone.0100592 (PMC4077703; doi:10.1371/journal.pone.0100592)
Supplement: Text S1 — Myelin Diffraction Analysis. Details of our analysis of the diffraction patterns are included in the sections titled: Observed and calculated intensities; Phase determination by replacement method; Diffracting power of myelinated fiber; Concentric multilamellar cylinders; and Surface structure and cylindrical averaged intensity calculation. (DOCX) [file pone.0100592.s005.docx]

**Text S1. Myelin Diffraction Analysis**

***Observed and calculated intensities***

Theory of myelin diffraction has been summarized previously [1], and is given in brief here. Assuming the apposition of like surfaces, the electron density $\rho(r)$ is centrosymmetric and given by$\rho(r)=\rho(-r)$. The Fourier transform of the unit cell electron density $\rho(r)$ is

$F( R )=2\int_{0}^{d/2} \rho(r)\cos(2\pi rR)dr$, where $R$ is the reciprocal coordinate in the same direction as the real coordinate $r$ in the stacking direction of the membranes, and the length is $\left|R\right|=2\sin\theta/\lambda$, where $2\theta$ is the scattering angle and $\lambda$ is wavelength. The structure factor contains only the real term, as the phase is either *0* or *π*. When the one-dimensional lattice has a period $d$, which corresponds to the myelin repeat distance of membrane pairs, $F(R)$ gives the values at $R=h / d$, where $h$ is an integer. The structure factor $F(h/d)$ is related to the observed intensity by a Lorentz correction $C(h)$ according to

$F_{obs}(h / d)=\pm\sqrt{C(h)I_{obs}(h / d)}$.

For the point-focus beam, the correction factor is *h^2^* . Using the observed structure amplitudes the electron density is calculated according to

$\rho(r)=(1 / d)\sum_{-\infty}^{\infty} F(h / d)\exp(-i2\pi rh / d)\approx F(0)/ d+(2 / d)\sum_{h=1}^{h_{\max}} KF_{obs}(h / d)\cos(2\pi rh / d)$,

where *K* is a scale factor. The structure factor at the origin $F(0)$ is given by $F(0)=\Delta F(0)+f d=d<\rho>_{d}=r_{0}<\rho>_{r_{0}}+f(d-r_{0})$, where $\Delta F(0)$ is the structure factor at the origin for the 'minus fluid model' [2, 3], $<\rho(r)>_{d}$ is the average of $\rho(r)$ in the range $\left|r\right|<d/2$, and $<\rho(r)>_{r_{0}}$ is the average of $\rho(r)$ in the range $\left|r\right|<r_{0}/2$, where $r_{0}$ is the exclusion distance, and $f$ is the electron density of the fluid medium. The absolute scale was derived previously by using a scale factor of 2.0, exclusion length 136 Å, average membrane electron density within the exclusion length 0.343 e/Å^3^, and fluid electron density 0.335 e./Å^3^ for swollen myelin in a hypotonic solution of glycerol [1, 4].

The continuous Fourier transform $F(R)$*,* which is the Fourier transform of $\rho(r)$ in the unit cell, is given by $F( R)=\sum_{-\infty}^{\infty} F(h / d) \sin c (\pi dR-\pi h)$.

The myelin swells and compacts under different condition which give rise to different periodicities. The observed structure amplitudes from the different data sets with different myelin periods were arbitrarily scaled according to $\sum\left|F_{obs}(h / d)\right|^{2} / d=0.03$.

***Phase determination by replacement method***

The electron density distribution of the unit cell is given by using the electron density of the asymmetric unit $\sigma(r)$ and the intra-unit, cell membrane pair separation *u* according to

$\rho(r)=\sigma(r)*\delta(r-u/ 2)+\sigma(-r)*\delta(r+u/ 2)$.

The Fourier transform of the asymmetric unit $\sigma(r)$ consists of symmetric and asymmetric terms, $F_{m}(R)=A(R)-iB(R)$; and the Fourier transform of the unit cell is written as

$$\begin{matrix} F_{u}(R)=[A (R)-iB (R)]\exp(i\pi uR)+[A (R)+iB (R)]\exp(-i\pi uR) \\ =2A(R)\cos(\pi uR)+2B(R)\sin(\pi uR). \end{matrix}$$

When the lattice period is $d$, the structure factor amplitudes are observed discretely only at $h / d$, where $h$ is an integer. The intensity is $[F_{u}(h / d)]^{2}=[2A ( h / d)\cos(\pi uh / d)+2B(h / d)\sin(\pi uh / d)]^{2}$.

If the asymmetric structure, myelin period, and packing distance are known, the phase of the structure amplitude is determined [5]. To derive the phase combination of the unknown structure, we first choose the known native myelin structure to obtain $A( R)-iB( R)$. Then the packing distance $u$ is derived by searching for the minimum R-factor between the observed and calculated structure amplitudes as a function of $u$ [6].

***Diffracting power of myelinated fiber***

The diffracting power (or the total intensity) is defined by . The structure factor for the exposed volume $v$ in the Cartesian coordinates is given by $F(X,Y,Z)=\int\int\int\rho(x,y,z)\exp[i2\pi(xX+yY+zZ)]dxdydz$, where $\rho(x,y,z)$ is the electron density distribution. The equatorial reflection at $(X,0,0)$ is

$F(X,0,0)=\int\int\int\rho(x,y,z)\exp(i2\pi xX)dxdydz=\int_{x} [\int_{y} \int_{z} \rho(x,y,z)dydz]\exp(i2\pi xX)dx$. This indicates that the $y,z$ projection of the electron density distribution along the $x$ axis is related to the equatorial intensity distribution. When the electron density is composed of two domains, one giving the periodical arrays along the $x$ axis and the other giving along other axes normal to *x*, the first crystalline domain will give the Bragg peaks in the $X$ direction. This diffracting power can be plotted as a function of the position of the incident x-ray beam. The relationship may be derived by using the size of the nerve fiber, the size of the axon, and the shape of the nerve’s cross-section (circle) (**Fig. S3**). For the circular shape a single myelinated nerve is assumed to consist of multi-concentric cylinders having outer radius $r_{0}$ and inner radius $r_{i}$. The axon is present within the region at $\left|r\right|<r_{i}$, and the bathing fluid is located at . The incident x-ray beam size is $2w$ and this is directed along the vertical direction (**Fig.** S**3**). The angle is assumed, so that the electron density distribution within the area ABCD gives the equatorial, lamellar Bragg reflections as a function of incident beam position $x>0$.

***Concentric multilamellar cylinders***

The area $S(x)$ as a function of the incident beam position $x$ for the concentric rings is considered. Different x-ray injection points are indicated by $p_{1},p_{2},p_{3},p_{4},p_{5},p_{6},p_{7},p_{8}$, where

The areas at different incident beam positions were then derived by

$S_{1}=0$ at $w<x\leq p_{1}$,

at $p_{1}<x\leq p_{2}$,

at $p_{2}<x\leq p_{3}$,

at $p_{3}<x\leq p_{4}$,

at $p_{4}<x\leq p_{5}$,

at $p_{5}<x\leq p_{6}$,

$S_{7}=2w [\sqrt{t_{3}}+\sqrt{t_{4}}]$ at $p_{6} <x\leq p_{7}$,

$S_{8}=r_{o}^{2}[\frac{\pi}{2}-a\sin\frac{v}{r_{o}}-\frac{1}{2}\sin2 (a\sin\frac{v}{r_{o}})]$ at $p_{7} <x\leq p_{8}$, and

$S_{9}=0$ at $x>p_{8}$, where

$$u=x+w, v=x-w, t_{1}=r_{i}^{2}-u^{2}, t_{2}=r_{i}^{2}-v^{2}, t_{3}=r_{o}^{2}-u^{2}, \text{and} t_{4}=r_{o}^{2}-v^{2}.$$

***Surface structure and cylindrical averaged intensity calculation***

The intensity distribution as a function of radial component of cylindrical coordinates $R$ was calculated using zero and first order of Bessel functions of the first kind ($J_{0}$ and $J_{1}$). This is given by $I(R)=F^{2}(R) Z(R)$, where $F( R)=r_{0} J_{1}(2\pi r_{0}R)/ R$ is the structure factor of a solid cylinder of radius $r_{0}$ and $Z(R)=\sum_{j} \sum_{k} J_{0}(2\pi r_{jk}R)$ is the interference where is the distance between the subunit vectors in cylindrical coordinates [7, 8]. A solid cylinder of a radius of 16 Å was used to represent the P0 extracellular domain. Solid cylinders (2, 3, and 4 in number) were placed on a circle of a radius of 28 Å [4]. The model calculation gave an intensity maximum similar to the observed one **(Fig. S2)**.

The cylindrically averaged intensity $<I(R,Z)>$, where $R$ and $Z$ are radial and axial components of the cylindrical reciprocal coordinates, was calculated using the atomic coordinates according to $<I( R,Z)> =[\sum_{j} \sum_{k} f_{j}( R,Z)f_{k}( R,Z) J_{0}(2\pi r_{jk}R )]\exp(i 2\pi z_{jk}Z)$, where $f(R,Z)$ is the atomic factor. In the current study the equatorial intensity at $Z=0$ was calculated. The intensity distribution for 2, 3, and 4 molecules, using the reported atomic coordinates determined by protein crystallography [9], showed diffuse scattering in the range of 0.015– 0.04 1/Å (**Fig. S2**). The intensity maximum at 40 Å was smaller than the observed spacing, indicating that the separation between the glycosylated full sequence P0-molecules in the whole nerve was larger by about 10 Å than the non-glycosylated extracellular domain of P0 that was analyzed by protein crystallography [9].

**REFERENCES**

1. Inouye H and Kirschner DA (2009) Myelin: A one-dimensional biological "crystal" for x-ray and neutron scattering. In: J. Sedzik and P. Riccio, editors. Molecules: Aggregation, Nucleation, Cyrstallization -- Beyond Medical and Other Implications. Singapore: World Scientific Publishing. pp. 75-94.

2. Worthington CR (1969) The interpretation of low-angle X-ray data from planar and concentric multilayered structures. The use of one-dimensional electron density strip models. Biophys J 9: 222-234.

3. Worthington CR (1971) X-ray analysis of nerve myelin. In: W. J. Adelman, Jr., editor. Biophysics and Physiology of Excitable Membranes. New York: Van Nostrand Reinhold Co., Inc. pp. 1-46.

4. Inouye H, Tsuruta H, Sedzik J, Uyemura K and Kirschner DA (1999) Tetrameric assembly of full-sequence protein zero myelin glycoprotein by synchrotron x-ray scattering. Biophys J 76: 423-437.

5. Caspar DL and Kirschner DA (1971) Myelin membrane structure at 10 A resolution. Nat New Biol 231: 46-52.

6. Kirschner DA and Ganser AL (1982) Myelin labeled with mercuric chloride. Asymmetric localization of phosphatidylethanolamine plasmalogen. J Mol Biol 157: 635-658.

7. Vainshtein BK (1966) DIffraction of X-Rays by Chain Molecules. Amsterdam: Elsevier Publishing Company.

8. Waser J (1955) Fourier transforms and scattering intensities of tubular objects. Acta Crystallogr 8: 142-150.

9. Shapiro L, Doyle JP, Hensley P, Colman DR and Hendrickson WA (1996) Crystal structure of the extracellular domain from P0, the major structural protein of peripheral nerve myelin. Neuron 17: 435-449.

10. DeLano WL (2002) The PyMOL Molecular Graphics System. San Carlos, CA: DeLano Scientific.
